# Metformin: A Narrative Review of Its Potential Benefits for Cardiovascular Disease, Cancer and Dementia

**DOI:** 10.3390/ph15030312

**Published:** 2022-03-04

**Authors:** Wiebe M. C. Top, Adriaan Kooy, Coen D. A. Stehouwer

**Affiliations:** 1 Department of Intensive Care, Treant Care Group, 7909 AA Hoogeveen, The Netherlands; w.m.top@treant.nl; 2 Department of Internal Medicine, Treant Care Group, 7909 AA Hoogeveen, The Netherlands; 3 Bethesda Diabetes Research Center, 7909 AA Hoogeveen, The Netherlands; 4 Department of Internal Medicine, University Medical Center Groningen, 9713 GZ Groningen, The Netherlands; 5 Department of Internal Medicine, Cardiovascular Research Institute Maastricht, Maastricht University Medical Center, 6202 AZ Maastricht, The Netherlands; cda.stehouwer@mumc.nl

**Keywords:** metformin, cardiovascular diseases, diabetes, cancer, dementia, pleiotropic effect

## Abstract

The biguanide metformin has been used as first-line therapy in type 2 diabetes mellitus (T2DM) treatment for several decades. In addition to its glucose-lowering properties and its prevention of weight gain, the landmark UK Prospective Diabetes Study (UKPDS) demonstrated cardioprotective properties in obese T2DM patients. Coupled with a favorable side effect profile and low cost, metformin has become the cornerstone in the treatment of T2DM worldwide. In addition, metformin is increasingly being investigated for its potential anticancer and neuroprotective properties both in T2DM patients and non-diabetic individuals. In the meantime, new drugs with powerful cardioprotective properties have been introduced and compete with metformin for its place in the treatment of T2DM. In this review we will discuss actual insights in the various working mechanisms of metformin and the evidence for its beneficial effects on (the prevention of) cardiovascular disease, cancer and dementia. In addition to observational evidence, emphasis is placed on randomized trials and recent meta-analyses to obtain an up-to-date overview of the use of metformin in clinical practice.

## 1. Introduction

Metformin, a synthetic biguanide, was first synthesized a 100 years ago, in 1922 [1]. It has been on the European market as an antidiabetic drug since 1958. However, competing glucose-lowering biguanides were regarded superior and marginalized the place of metformin. When the competing biguanides, especially phenformin, were withdrawn in the late 1970s due to lactic acidosis as a serious side effect, metformin remained the only available biguanide on the market.

It took another 25 years before metformin was allowed on the US market in 1995. This was due to concerns about the safety profile, especially the occurrence of lactic acidosis. To further strengthen the safety profile of metformin, the FDA required a post-marketing safety surveillance study, which was published in 2005. This Comparative Outcomes Study of Metformin Intervention vs. Conventional Approach (COSMIC) showed no difference in serious side effects between metformin and conventional therapy and, in particular, no single case of lactic acidosis [2].

In the meantime, in 1998, the United Kingdom Prospective Diabetes Study (UKPDS) had been published showing that metformin, in addition to lowering the blood glucose, reduced cardiovascular mortality in obese T2DM patients. This finding was further strengthened by the 2008 publication of the 10-year follow of the UKPDS, which showed that significant reductions persisted in the metformin group for any diabetes-related endpoint (risk reduction (RR) 0.79, 95% confidence interval (CI) 0.66–0.95), myocardial infarction (RR 0.67 95% CI 0.51–0.89) and death from any cause (RR 0.73 95% CI 0.59–0.89) [3].

Based on a steadily increasing evidence base of positive effects of metformin coupled with a good safety profile, its prevention of weight gain and low cost, metformin has become the most widely prescribed drug in the treatment of type 2 diabetes mellitus (T2DM) and is recommended in European and American guidelines as first-line therapy in T2DM [4].

However, its leading position is now being debated since two new drug classes with strong cardiovascular protective properties have come on the market. This concerns sodium-glucose cotransporter 2 (SGLT2) inhibitors and glucagon-like peptide 1 (GLP-1) receptor agonists. Given the robust evidence of these new drug classes in large cardiovascular outcome trials, the place of metformin is being reassessed.

Apart from cardiovascular benefits, there is growing evidence that metformin may be beneficial for other age-related diseases, such as cancer, cognitive impairment and dementia. Additionally, metformin has been studied in various other diseases, such as polycystic ovary syndrome (PCOS), osteoporosis, periodontitis, inflammatory bowel disease, non-alcoholic fatty liver disease, COVID-19 and as anti-aging drug in general [5].

Thus, the field of application for metformin has broadened instead of diminished in recent decades, and metformin is being repurposed as a geroprotective agent that may promote healthy aging and extend lifespan. This review will give an update of the available evidence of the beneficial health outcomes of metformin regarding cardiovascular disease, cancer, cognitive impairment and dementia, both in the diabetic population and the non-diabetic population.

## 2. Metformin

### 2.1. Biguanides

Biguanides are known to have a wide variety of therapeutic indications. Among others they are used as antidiabetic, antimalarial, anticancer, antimicrobial and antiviral agents [6]. From all biguanides, most research has been done with metformin.

The first clinical application of metformin was as anti-flu agent in malaria patients by Garcia in 1950 [7]. The glucose-lowering effect was noticed by Garcia and regarded as a possible mechanism by which metformin could destroy malaria parasites. It was Sterne who changed the paradigm and proposed metformin as a primary antidiabetic agent in 1957 [8].

The most relevant side effect of biguanides is mitochondrial toxicity leading to lactic acidosis. Although biguanides inhibit isolated mitochondrial complex 1, only those biguanides that enter mitochondria do so in vivo. Biguanide-associated activation of AMP-activated protein kinase (AMPK), which results in glucose lowering, also is related to access to the mitochondrial compartment [9]. This is the reason that some biguanides do possess glucose-lowering properties but also major toxicity. Metformin seems to touch the sweet spot with minimal toxicity and effective AMPK activation.

### 2.2. Glucose Lowering Mechanisms

Metformin is a hydrophilic cation with metal-binding properties, especially with copper. Because of its hydrophilic character, it is not able to diffuse through lipid-rich membranes and needs a transporter. Organic cation transporter 1 (OCT1) is an important metformin transporter and the OCT1 expression of cells affects the uptake of metformin. Polymorphisms of this transporter however do not influence the glycemic response to metformin [10].

Oral bioavailability of metformin is between 50 and 60%. Apart from the kidneys and the bladder which dispose unmetabolized metformin, increased concentrations of metformin are primarily found in the intestinal and colonic wall and the liver with tissue-to-plasma ratios of 300 and 3, respectively [11].

The main glucose-lowering property of metformin is a reduction in the fasting blood glucose. Historically this has been ascribed to a reduction in hepatic gluconeogenesis and hence a reduction in hepatic endogenous glucose production. Especially in long-duration T2DM, this is an important mechanism since increased hepatic glucose output is a well-known pathophysiological mechanism [12].

However, a systematic review showed that reduced endogenous glucose production only explained part of the reduction in fasting glucose and that increased glucose disposal is another major part [13]. This has been further corroborated by recent studies showing that, in recent-onset T2DM, metformin may even increase endogenous glucose production [14].

The major site of action regarding glucose disposal may be the intestinal tract. Metformin restores glucose uptake in the small intestine and colon. A 26-week, randomized, placebo-controlled trial evaluated the intestinal glucose uptake with fluorodeoxyglucose positron-emission tomography (FDG-PET) imaging, showing a 2-fold increase in the small intestine and 3-fold increase in the colon in metformin users [15]. In addition, metformin shifts the gut microbiota composition and stimulates GLP-1 secretion by enterocytes as well as the sensitivity for GLP-1 [16]. These are additional mechanisms, because the intestinal glucose transport effects of metformin are independent of GLP-1 secretion [17].

An important intestinal site of action is also in accordance with studies that showed that metformin was as effective with delayed absorption targeting the ileum, as compared with immediate resorption. The glycemic effects of metformin persisted despite lower plasma metformin levels in the delayed absorption group [18]. Additionally, intravenous administration of metformin showed no acute changes in glucose metabolism, in contrast with oral administration [19].

Additionally, the side effects of metformin are related to the gastrointestinal tract. Up to 30% of metformin users experience nausea, vomiting, diarrhea, bloating or abdominal pain [20]. An impaired ileal absorption of bile salts contributes to osmotic bile salt diarrhea. Nausea, taste aversion and decreased appetite may be related to increased secretion of growth-differentiation factor 15 (GDF-15) by enterocytes [21].

Apart from the liver and the intestines, increased glucose uptake in skeletal muscle has been reported as an additional glucose-lowering mechanism [22].

### 2.3. Cellular Mechanisms

The pleiotropic effects of metformin, transgressing the boundaries of T2DM, may be explained by several cellular mechanisms which have been discovered during the last decades. Central in the cellular working mechanism of metformin is the mitochondrion. Metformin is able to modulate mitochondrial function and hence the bioenergetic status of the cell. In hypermetabolic states metformin may favorably lower mitochondrial respiration, while in hypometabolic states or resting conditions, mitochondrial respiration may be enhanced [23].

Apart from being the major energy source, mitochondria also play a key role in apoptosis and the production of reactive oxygen species (ROS). By modulating mitochondrial ion channels, metformin can reduce apoptosis in neuronal cells [24]. Inhibition of ROS leakage from mitochondria to the cell may be an additional important mechanism by which metformin is able to attenuate myocardial ischemia–reperfusion injury [25,26] and vascular injury [27], and prevent carcinogenic DNA mutations [28].

In the following sections we will discuss some main mechanisms by which metformin exerts its effects (Table 1). However, a single and universal working mechanism has not been found; instead, there are multiple parallel pathways which may or may not be present depending on the cell type, the presence of transporters, the metformin dose, the duration of the exposure and the metabolic background.

### 2.4. Mitochondrial Pathways

**Figure 1:**
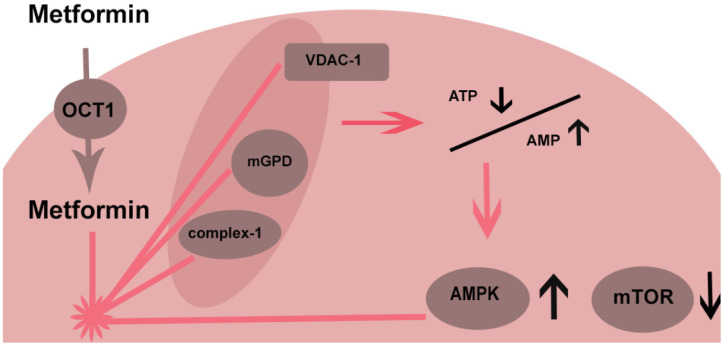
AMPK—AMP protein kinase; OCT—organic cation transporter; mGPD—mitochondrial glycerophosphate dehydrogenase; VDAC—voltage-dependent anion channel; mTOR—mammalian target of rapamycin.

There are several molecular pathways by which metformin may modulate mitochondrial function (Figure 1). One of these is inhibition of respiratory complex type 1. This has been shown in vitro using millimolar metformin concentrations [45]. Inhibition of complex 1 leads to a cascade of decreased ATP, increased AMP and decreased cyclic AMP. These alterations in turn activate AMPK, which will be discussed below.

This inhibition of respiratory complex 1 may apply in the enterocyte in which high metformin concentrations are present. However, tissue concentrations in the liver are much lower. Because of the negative inner mitochondrial membrane charge, such high metformin concentrations may theoretically be achieved in hepatic mitochondria, but this remains to be proven [46].

Apart from inhibition of complex 1, metformin may influence the mitochondrial glycerophosphate shuttle altering the cytosolic redox state [12]. This concept explains the selective inhibition of gluconeogenesis of redox-dependent substrates (lactate and glycerol) as opposed to non-redox-dependent substrates. This proposed mode of action has been criticized as an explanation for a decrease in gluconeogenesis because the other important redox shuttle, the malate–aspartate shuttle, would be expected to compensate.

Another possible mitochondrial target is the voltage-dependent anion channel (VDAC-1). Metformin is able to displace hexokinase from these channels, limiting cytoplasmal access to mitochondrial ATP and hence limiting the energy supply of the cell [47]. Depending on the type of hexokinase, some cells such as cancer cells are susceptible to metformin inhibition and others, such as hepatocytes, are not affected [37]. VDAC1 also functions as an apoptotic protein release channel by which metformin may protect against release of apoptotic proteins to the cytoplasm by modulating the anion channel.

Although mitochondrial pathways are important, metformin also has effects that are mediated by other pathways. For example, metformin has effects on membrane fluidity and glucose metabolism in erythrocytes (which do not contain mitochondria) [48].

### 2.5. AMPK

AMPK is a key regulator in modulating the bioenergetic status of the cell. Metformin is an activator of AMPK. AMPK, as a cellular energy sensor, is activated by a decrease in ATP and an increase in AMP. Metformin could activate AMPK by inhibiting mitochondrial respiration and hence lower ATP levels, but other pathways have been reported to be involved.

For example, metformin is able to increase AMP levels by slowing down the degradation of AMP [49]. Additionally, lysosomal pathways have been reported that activate AMPK [50].

Activation of AMPK results in an energy conserving state (analogous to caloric restriction) in which ATP-consuming anabolic pathways such as gluconeogenesis are switched off. Simultaneously a downstream cascade of signaling molecules is activated promoting a catabolic state with glycolysis and oxidation of fatty acids to restore the energy balance.

The inhibition of anabolic pathways is not restricted to gluconeogenesis and also applies to lipogenesis, proteins and ribosomal RNA. An important enzyme which is inhibited by metformin is mammalian target of rapamycin (mTOR).

This multiprotein complex is a major anabolic regulator that has a role in the metabolism and proliferation of malignant cells which is an important link to potential anticancer effects of metformin. mTOR inhibition is both regulated through AMPK activation but in AMPK knockout mammalian cells also AMPK-independent pathways inhibiting mTOR have been shown to exist [51].

### 2.6. AMPK-Independent Effects

In addition to AMPK-mediated effects, metformin also has AMPK-independent actions. It was shown that metformin is able to inhibit gluconeogenesis in AMPK-knockout mice [52]. To further complicate matters, some downstream effects of metformin may both be regulated independent of AMPK and as consequence of AMPK activation.

In AMPK-knockout mice, metformin was able to decrease gluconeogenic flux without direct inhibition of gluconeogenic gene expression (as AMKP activation would do). This might be explained by allosteric regulation of gluconeogenic key enzymes such as phospho-fructokinase. Therefore, activation of AMPK may be a secondary mode of regulation that is sufficient but dispensable [53].

This applies also to the anti-inflammatory properties of metformin. Metformin is associated with a reduction in several pro-inflammatory mediators, among which are the master regulator nuclear factor kappa B (NF-kB), interleukin 6 and tumor necrosis factor alpha [54]. This effect was independent of the presence of AMPK [55]. Chronic inflammation underlies both endothelial damage and carcinogenesis. Therefore, metformin may ameliorate these processes by limiting inflammation.

Tumor cells display an increased metabolism in which copper acts as a cofactor. Regarding the metal-binding properties of metformin, the binding of copper may be important. Binding of mitochondrial copper by metformin will slow down the mitochondrial electron transport chain and inhibit the differentiation of tumor cells from the epithelial to the metastatic prone mesenchymal state [38]. Additionally, metformin–copper complexes may bind intracellular antioxidants, making the cell more susceptible to ROS damage and apoptosis [56].

Lastly, lactate as an alternative metabolic substrate may underlie some of the beneficial effects of metformin. Already damaged organs especially, such as diabetic heart, kidney and neurons, have reduced capacity for glucose utilization and lactate may fill the gap. Metformin stimulates intestinal lactate production. Provided that toxic metformin concentrations are avoided, the physiological elevation of lactate may be beneficial rather than harmful [33].

## 3. Cardiovascular Effects

Several meta-analyses evaluating the effect of metformin on cardiovascular outcomes have been performed, but, depending on the selection criteria, few adequate randomized trials could be included in those meta-analyses.

Since 2006, metformin is considered first-line therapy in the consensus statement of the American Diabetes Association (ADA, Arlington County, VA, USA) and the European Association for the Study of Diabetes (EASD, Montecatini Terme, Italy). This recommendation was mainly based on the UKPDS study awaiting further confirmation. Instead of further confirmation trials comparing metformin with placebo or with an active comparator, most large cardiovascular outcome trials have been performed with new classes of medication (SGLT2 inhibitors, GLP1 receptor agonists), mostly on top of metformin.

Recent meta-analyses of randomized controlled trials with metformin mostly concluded an absence of evidence or a neutral effect regarding beneficial effects of metformin on cardiovascular outcomes (Table 2) [57,58,59,60]. However, the available evidence is weak because most studies were poorly conducted [60]. With regard to beneficial cardiovascular effects of metformin, we will discuss three trials that are often quoted.

### 3.1. UKPDS

The first is the UKPDS trial of 1998 for which the 10-year follow-up results were published in 2008 [3]. The main comparison was between intensive treatment (glycemic goal fasting plasma glucose (FPG) < 6 mmol/L) vs. conventional treatment (FPG < 15 mmol/L) and the median follow up was more than 10 years at the time of the first publication.

Compared with current guidelines, the glycemic treatment goals for the conventional treatment group and the standard of care are outdated and reflect suboptimal treatment. Treatment options apart from diet were insulin or sulfonylurea (SU) for non-obese persons with the addition of metformin for obese persons. Because the intensive treatment goal was hard to achieve with monotherapy, there were many cross-over patients who were prescribed combination therapy. The main analysis was however an intent to treat analysis.

The pre-specified obese subgroup of 1704 patients in which metformin (*n* = 342) was randomized vs. diet (*n* = 411) showed risk reductions of 32% (95% CI 13–47) for any diabetes-related endpoint, 42% for diabetes-related death (95% CI 9–63), and 36% for all-cause mortality (95% CI 9–55) [61].

Compared with other intensive therapies (insulin or SU), metformin performed better for any diabetes-related endpoint, all-cause mortality and stroke. This means that the effects found are not only related to improved glycemic control (which the other intensive therapies also achieved) but are related to non-glycemic mechanisms either caused by beneficial effects of metformin or by detrimental effects of the comparator therapy.

A substudy that was performed within the UKPDS in which addition of metformin to patients who failed on SU monotherapy was compared with staying on SU showed an increased mortality for patients who were randomized to metformin. This was an unexpected finding for which the authors performed an extra analysis in the complete UKPDS study population to evaluate the combination of metformin and SU. No firm conclusions could be drawn regarding possible harm and further study was advised. Later follow-up data showed that the increased mortality in this group subsided over time; in retrospect, it was regarded as a chance finding [65].

In contrast, the beneficial effects of metformin turned out to be robust and lasting as shown in the 2008 follow-up publication. In the metformin group, significant risk reductions persisted for any diabetes-related endpoint, myocardial infarction and death from any cause [3].

### 3.2. HOME

The second trial is the 2009 “Hyperinsulinemia: The Outcome of its Metabolic Effects” (HOME) trial [62]. This placebo-controlled randomized trial with metformin as add-on therapy in 390 insulin-using T2DM patients, not using other antihyperglycemic agents, had a follow up of 4.3 years. The primary endpoint of a combined micro- and macrovascular aggregate was not reached but the prespecified secondary macrovascular aggregate score was improved in the metformin group (hazard ratio (HR) 0.61 95% CI 0.40–0.94).

Despite randomization, patients in the metformin group were slightly older and had a more extensive cardiovascular history. Therefore, in the statistical analysis, the crude results were adjusted for these baseline differences.

The macrovascular aggregate score included both mortality and cardiovascular morbidity. The beneficial effect of metformin was primarily observed in reduced manifestations of cardiac ischemia. Absolute mortality however was greater in the metformin group compared with placebo. Because meta-analyses often use crude mortality or crude cardiovascular events, they use individual components of the secondary endpoint which does not reflect the combined macrovascular aggregate score.

There have been several additional analyses of the HOME trial focusing on cardiovascular aspects. It was shown that metformin was associated with improvement in some markers of endothelial function [32]. With regard to specific cardiac effects, metformin did not affect N-terminal pro B-type natriuretic peptide (NT-proBNP) plasma levels [66].

### 3.3. SPREAD-DIMCAD

The third trial is the 2013 “Study on the Prognosis and Effect of Antidiabetic Drugs on T2DM with Coronary Artery Disease” (SPREAD-DIMCAD) [63]. This trial included 304 T2DM patients with a history of CAD with a median follow up of 5 years. Patients were randomized between metformin or an sulphonylurea.

The primary endpoint was time to a macrovascular aggregate score including death from any cause and myocardial infarction. The hazard ratio of the metformin users compared with the SU group for the primary endpoint was 0.54 (95% CI 0.30–0.90).

A strength of this study is that the effects of SU treatment and metformin can be assessed separately. In this population of high-risk patients, the benefit of metformin compared with SU was shown, although there was no comparison with placebo. Therefore, the effects could be both explained by beneficial effects of metformin or by deleterious effects of the SU treatment.

### 3.4. Other Relevant Trials and Meta-Analyses

An example of a large randomized controlled trial (RCT) in which an opposite trend was shown is the 2006 “A Diabetes Outcome Progression Trial” (ADOPT) study [67]. In this active comparator study, over 4000 T2DM patients were included and randomized between thiazolidinediones (TZD), SU and metformin. Death from any cause did not differ between treatment groups, but cardiovascular events had a lower incidence in the SU group; however, the study was not powered to assess cardiovascular events.

Another trial which is used in meta-analyses is a trial in which contraindications of metformin were evaluated [68]. In this trial, patients were randomized between more conservative vs. more liberal contraindications, according to which metformin was stopped or continued. Continued use of metformin in the liberal group compared to stopping metformin in the conservative group did not result in different cardiovascular outcomes within a follow up of 4 years. However, there may be a long-term beneficial metformin effect in the conservative group despite stopping the drug (legacy effect), as shown by UKPDS.

A recent summary of the available cardiovascular evidence regarding metformin was provided by the Italian Society of Diabetology and the Italian Association of Clinical Diabetologists [64]. They published an updated meta-analysis in 2021 in which they only selected the UKPDS and the SPREAD-DIMCAD for their endpoint on major cardiovascular effects with a combined odds ratio of 0.52 (95% CI 0.37, 0.73) favoring metformin and a non-significant trend for reduced all-cause mortality (odds ratio (OR) 0.80 (95% CI 0.60, 1.07)).

With regard to the all-cause mortality endpoint, over 90% of the weight of their meta-analysis was composed of the UKPDS, the HOME, the SPREAD-DIMCAD and the ADOPT trials. The authors underline the small sample size, the need for a long follow up to assess mortality rates and the paucity of placebo-controlled studies. Active comparator studies induce altered cardiovascular risk in the comparator arm. A decrease in all-cause mortality in metformin users became significant when active comparator studies were excluded.

Another recent meta-analysis had a broader scope and included both metformin monotherapy and combination therapy including over 7000 patients [69]. Although micro- and macrovascular events both had a decreasing trend in metformin users, statistical significance was not reached. Compared with drug regimens without metformin, the risk of all-cause mortality and cardiovascular mortality was increased in metformin combination therapy. However, the UKPDS substudy evaluating the combination of sulfonylurea and metformin was the major contributor to this analysis and the interpretation of this substudy is controversial as the UKPDS authors later described the increased mortality to chance, as mentioned before.

### 3.5. Observational Data

In contrast to the paucity of well-designed randomized trials, there is an overwhelming amount of observational data. A recent meta-analysis of cohort studies included over a million patients and showed a strong beneficial effect of metformin with an odds ratio of 0.57 (95% CI 0.48–0.68) for combined mortality and cardiovascular events compared with T2DM patients treated with other drugs [70].

Another observational study concerns a meta-analysis which includes comparison with non-diabetic individuals. This analysis shows a reduced risk for all-cause mortality for metformin users compared with non-diabetic individuals (HR 0.93, 95% CI 0.88–0.99). Compared with diabetes patients receiving non-metformin therapy the risk reduction was stronger (HR 0.72, 95% CI 0.65–0.80) [71].

Additionally, analyses in high-risk patient populations show benefit of metformin therapy. A meta-analysis in diabetes patients with established coronary artery disease showed a reduction in cardiovascular mortality (HR 0.81 95% CI 0.79, 0.84), all-cause mortality (HR 0.67 95% CI 0.60, 0.75) and the incidence of cardiovascular events (HR 0.83 95% CI 0.78, 0.89) [72].

Another meta-analysis in patients with T2DM and chronic kidney disease (stage G1–G3) showed a reduction in all-cause mortality (RR 0.71, 95% CI 0.61, 0.84) and the incidence of cardiovascular events (RR 0.76, 95% CI 0.60, 0.97) [73].

### 3.6. SGLT2 Inhibitors and GLP-1 Receptor Agonists

Since the introduction of new classes of medication for the treatment of T2DM, there have been large cardiovascular outcome trials that showed the benefits of both SGLT2 inhibitors and GLP1 receptor agonists. The cumulative evidence for cardiovascular risk reduction in the new agents is more robust than the available evidence of metformin. However, given the fact that metformin is still first-line therapy, most (60–82%) of these trials have been performed with metformin as baseline therapy [74]. Although subgroup comparisons and meta-analyses (metformin vs. non-metformin) have been made, patients were not randomized for metformin use and the reason for not having metformin at baseline (side effects, contraindications) may introduce bias.

Notwithstanding the proven cardiovascular benefits of GLP-1 receptor agonists and SGLT-2 inhibitors, the additional benefits of metformin as baseline therapy have not been disproved. Additional head-to-head comparisons between metformin and GLP-1 receptor agonists and SGLT-2 inhibitors in a large, randomized trial are not to be expected and neither are cardiovascular outcome studies with metformin vs. placebo.

Therefore, we have to make decisions based on our current evidence base. This evidence still fits with the position of metformin as baseline therapy combined with new drug classes for high-cardiovascular-risk patients, instead of competing with them. To trade in metformin as baseline therapy for new drug classes would deny the patient any additional benefits of metformin therapy. Because metformin does not increase the risk of hypoglycemia, it is very well suited for combination therapy.

## 4. Cancer

People with T2DM have a higher risk of cancer and increased mortality rates due to cancer compared with those without diabetes. Although cardiovascular disease used to be the most important cause of diabetes-related death, there has been a transition to cancer as leading cause in high-income countries [75].

A recent epidemiological analysis showed a 30% decline in all-cause death rates over the last 15 years in the UK [75]. Although both cardiovascular death rates and cancer death rates declined, dementia as a cause of death increased and absolute mortality rates of cancer surpassed the cardiovascular rates. Despite improvements in mortality rates, the gap between individuals with and without diabetes remained.

The same pattern was seen in a Hong Kong study, in which cancer rates also surpassed the cardiovascular death rates in patients with diabetes [76]. In this study, pneumonia was the leading cause of death. This may well be, in part, a proxy for dementia, since classification systems vary between countries.

In 2014, an umbrella review was published of meta-analyses from observational data regarding the association between T2DM and the risk of developing or dying from certain forms of cancer [77]. The most robust evidence was seen for breast cancer, intrahepatic cholangiocarcinoma, colorectal cancer and endometrial cancer (Table 3). The authors emphasize that despite not passing their exclusion criteria for possible bias, strong associations are also found for hepatocellular and pancreatic cancer.

Generic mechanisms in diabetes, such as hyperglycemia and the resulting hyperinsulinemia with concurrent increased free IGF-1, might be harmful and contribute to the formation and growth of malignant tumors [34,35]. Metformin, as an insulin-sensitizing agent, will decrease insulin and free IGF-1 levels.

In addition to these indirect effects, metformin has multiple drug-specific effects with regard to cancer. These include the effects of metformin as AMPK activator with resulting mTOR inhibition. However, also a decrease in inflammation and oxidative stress, and a reduction in glycolysis, which is used by many rapidly growing tumors as preferred metabolic route [37]. Metformin may also enhance the immune response against tumors by modulating immune cells in the tumor microenvironment [39].

An observational study in Scotland compared median time to cancer diagnosis in 4000 metformin users compared to 4000 non-metformin users with T2DM. This study showed an unadjusted hazard ratio of 0.46 (95% CI 0.40, 0.53) for the incidence of cancer. After adjustment the hazard ratio was 0.63 (95% CI 0.53, 0.75) [78]. An overview of studies with metformin performed in the last 5 years showed a possible protective effect in the incidence of colorectal, hepatic, head and neck and lung cancers [79].

A Chinese meta-analysis with more than 10 million T2DM patients showed an overall reduction of 20% in cancer incidence in metformin users compared with other diabetes drugs (OR 0.80, 95 CI 0.73–0.87) with in subgroup analysis a decreased risk in bladder, colorectal, gastric, liver, lung, pancreatic and prostate cancer [80]. The strongest risk reductions compared with other diabetes drugs were reported for gastric cancer (HR 0.53 95% CI 0.42–0.65), pancreatic cancer (HR 0.57 95% CI 0.35–0.93) and liver cancer (HR 0.62 95% CI 0.44–0.89).

In the following section we will discuss the effect of metformin on the three major cancers worldwide: breast, colon and lung cancer (Table 4).

### 4.1. Breast Cancer

Breast cancer was the most prevalent cancer worldwide in 2020, with over 2 million new cases. The risk of breast cancer is increased in T2DM. As most cases of breast cancer are estrogen-sensitive, the hormone receptor status of the tumor could influence treatment outcomes.

Estrogen-receptor-positive (ER+) tumors are the most frequent type of breast tumor (80%). The hormone receptor status of breast tumors differs between T2DM patients and non-diabetic patients with breast cancer. Diabetes patients especially on metformin therapy tend to have less ER+ tumors and instead more ER− and even triple negative tumors [86]. Possibly by protecting against ER+ tumors, a selection of ER− and triple negative tumors remains.

Apart from improving insulin sensitivity, decreasing glucotoxicity and activating AMPK with multiple downstream pathways, metformin decreases estradiol levels with roughly 30% independent of body mass index [36]. This could be an additional pathway by which metformin could favorably slow down tumor growth in estrogen-sensitive tumors.

The effect of metformin on breast cancer remains controversial. Observational data, while showing mixed results, do support a protective effect of metformin on breast cancer incidence. An example is a Taiwanese retrospective study of almost 500,000 patients showing a reduced incidence rate of breast cancer among metformin using T2DM patients with a rate reduction from 5 per 1000 person-years in non-metformin users compared with 2 per 1000 person-years in metformin user [81].

An observational cohort study in Scotland showed, in the subgroup of breast cancer patients, an unadjusted hazard ratio of 0.44 (95% CI 0.26–0.73) and an adjusted ratio of 0.60 (95% CI 0.32–1.10) for incident cancer in metformin users [78]. A recent Chinese prospective observational study of almost 4000 patients with incident breast cancer showed significant survival differences favoring metformin. In contrast, the use of insulin was associated with decreased survival compared with the non-diabetes group [83].

With regard to randomized trials there are conflicting results. A recent meta-analysis of randomized trials evaluated a mixed outcome of tumor growth, apoptosis and biomarkers in 1600 metformin-treated non-diabetic female breast cancer patients [87]. It showed a reduction in the pooled hazard rate of the various endpoints (HR 0.63, 95% CI 0.59–0.71).

However, randomized trials in phase 2 trials fail to show benefit of metformin. In non-diabetic patients, no survival effect of metformin in metastatic/recurrent breast cancer was seen when used as adjunctive therapy [88,89,90].

In summary, there is strong observational evidence that metformin may reduce the incidence of breast cancer, in particular estrogen-positive tumors [78,81,86]. Additionally, in patients diagnosed with breast cancer, metformin was associated with lower risk of a second breast tumor, recurrence or breast-cancer-related death [82,83]. However, randomized trials in metastatic disease and recurrent breast cancer fail to show a benefit of metformin as adjunctive therapy [88,89].

### 4.2. Colorectal Cancer

Colorectal cancer (CRC) was the third most incident cancer worldwide and the second most common cause of cancer-related death in 2020. Patients with T2DM have an increased risk of CRC and related death.

Three recent meta-analyses of observational data confirm a beneficial effect of metformin on the incidence of CRC, all-cause mortality of CRC and lower CRC-specific mortality. An 2021 meta-analysis of 28 studies including over 2 million people showed that the use of metformin reduced the risk of CRC by 29% compared with nonuse (OR/RR = 0.71, 95% CI, 0.64–0.80) [84]. This finding was in line with two 2020 meta-analyses which found a reduced incidence of CRC in diabetic patients of 12% (adjusted RR 0.88, 95% CI 0.83–0.94) [91] and 24% (RR 0.76, 95% CI 0.69–0.84) [92].

Although most of these meta-analyses were based on observational data, there has been a randomized trial in patients without diabetes on the recurrence of colon adenoma or polyps after resection of an adenoma [93]. A total of 151 patients were randomized between low-dose metformin (250 mg) or placebo and underwent a 1-year follow-up colonoscopy. The prevalence of polyps and adenomas was reduced in the metformin users (RR 0.67 95% CI 0.47–0.97 and RR 0.60 95% CI 0.39–0.92, respectively).

With regard to prognosis of CRC patients, an all-cause mortality benefit was reported of 28% (HR 0.72, 95% CI 0.62–0.83) and CRC-specific mortality of 20% (HR 0.80, 95% CI 0.70–0.92) [84]. Another meta-analysis reported reduced all-cause mortality rates of 40% (HR 0.60, 95% CI 0.53–0.67) and CRC specific rates of 34% (HR 0.66, 95% CI 0.59–0.7) [92].

Most of the data are again observational but there have been phase 2 studies. In treatment refractory CRC, 41 patients were treated with the combination of metformin and irinotecan. From other placebo-controlled studies the placebo response rate in this setting was 13% after 12 weeks. In this study 41% reached disease control after 12 weeks, consistent with a beneficial effect of metformin [94].

Another phase 2 study, including 50 patients with refractory metastatic CRC, studied the combination of 5-fluorouracil and metformin. Although the predefined response rate of 20% after 8 weeks was met in the metformin group (22%), it was only a modest response [95].

Apart from potential synergistic effects with chemotherapy, metformin is also used as radiosensitizer. Two clinical trials demonstrated an improved tumor and nodal downstaging and pathological complete response rates for rectal cancer using metformin [96,97].

Hence, there is strong observational data supported by one randomized trial that metformin is able to reduce the incidence of CRC. Concerning benefits for the treatment of CRC observational data also suggest survival benefits; however, randomized trials are lacking, and phase 2 trials may only indicate a small benefit.

### 4.3. Lung Cancer

Lung cancer was the leading cause of cancer-related death in 2020 and the second most common cancer. Other than breast and colon cancer, its incidence is not increased in diabetes so beneficial effects of metformin cannot be ascribed to generic diabetes treatment effects.

A 2020 meta-analysis evaluated the incidence of lung cancer and the use of metformin in 8 observational studies with over 200,000 patients with T2DM. This study found a decreased risk of lung cancer in metformin users (HR 0.78; 95% CI 0.70–0.86) [85].

Two recent meta-analyses combined the observational data and the available randomized trials on the role of metformin in lung cancer survival. In one meta-analysis, the observed overall survival improved both in studies without a time-dependent approach (HR 0.74, 95% CI 0.60–0.91), as well as in studies using a time-dependent approach (HR 0.84, 95% CI 0.79–0.88) [98]. In the other meta-analysis, the combined overall survival for observational studies and randomized trials was HR 0.74 (95% CI 0.68–0.81) [99]. In addition, the progression-free survival was also improved with a HR of 0.81 (95% CI 0.74–0.88).

Three randomized trials of metformin use in non-small cell lung cancer (NSCLC) non-diabetic patients taken together did not reach statistical significance but showed a trend towards improved survival in the two meta-analyses (HR 0.86, 95% CI 0.68–1.08 and HR 0.73, 95% 0.47–1.15).

One of the included trials randomized 139 non-diabetic lung cancer patients between epidermal growth factor receptor (EGFR)–tyrosine kinase inhibitors and the addition of metformin [100]. Both the primary endpoint of progression-free survival and the secondary endpoint of overall survival improved in the metformin users (HR 0.60; 95% CI 0.40–0.94 and HR 0.52 95% CI 0.30–0.90, respectively).

A post hoc analysis of this study was published this year in which the effect of body mass index (BMI) on the protective effect of metformin was evaluated. Only patients with BMI > 24 showed beneficial effects of metformin. This could be an explanation for the heterogeneity of the results of the randomized trials, as the overweight rate in Asian populations is markedly lower than that in Hispanic populations [101].

In summary the evidence for a beneficial effect of metformin on both the incidence and the treatment of lung cancer is convincing although data from randomized trials are still lacking behind observational data.

## 5. Cognitive Function

In contrast to cardiovascular disease and cancer, the incidence of dementia is rising, and in some countries, it is the most common cause of death (depending on the classification scheme used) [75].

The most invalidating and common form of dementia is Alzheimer’s disease. This is an age-related disease which usually starts in individuals of 65 years and older.

With regard to the pathogenesis of Alzheimer’s disease, the most accepted model is the amyloid hypothesis. This model is based on the typical pathological findings of β-amyloid plaques and neurofibrillary tangles composed of abnormal tau protein. However, the metabolic processes leading to the formation of these plaques and tangles remain poorly understood [102].

Both microvascular disease and insulin resistance are linked to cognitive dysfunction either initiating, complicating or accelerating the disease process. Although obesity and insulin resistance worsen cognitive function, they do not lead to the typical pathological hallmarks of Alzheimer’s dementia. Instead, an independent form of insulin resistance confined to the brain is proposed as an additional phenotype of diabetes (type 3 diabetes) [41,103].

Because of this metabolic background, diabetes treatment may slow down and reduce the formation of amyloid and neurofibrillary tangles. In animal research, metformin has been shown to reduce amyloid formation [44]. In addition to improving insulin sensitivity, metformin may also provide endothelial protection [32]. Therefore, microvascular damage resulting in cognitive dysfunction may be reduced.

In addition, hypoglycemia is an independent risk factor for dementia [104,105]. Treatment with glucose lowering pharmacotherapy has an inherent risk for iatrogenic hypoglycemia, especially in older T2DM patients with a long diabetes duration treated with insulin or sulfonylurea [106]. By contrast, metformin has a low risk for hypoglycemia.

Several meta-analyses have been performed the last years evaluating the role of metformin in modulating neurodegenerative disease. A 2018 meta-analysis reported a decreased risk for cognitive impairment in T2DM patients using metformin compared with non-metformin users (OR 0.55 95% CI 0.38–0.78) and a decreased risk for dementia (OR 0.76 95% CI 0.60–0.97) [107].

However, an updated meta-analysis in 2020 comprising more trials concluded a neutral effect (OR 1.04 95% CI 0.92–1.17) for overall neurodegenerative disease and in a subgroup analysis also a neutral effect for dementia (OR 0.96 95% CI 0.85–1.08) [108]. In addition, there was an increased risk of Parkinson’s disease (PD) in the metformin group (OR 1.66 95% CI 1.14–2.42). This increased risk of PD in metformin users was also found in a dedicated meta-analysis to assess the risk of antihyperglycemic drugs on the incidence of PD in diabetes patients [109].

A combined network meta-analysis and meta-analysis, also published in 2020, evaluated over 1 million patients and assessed the effect of various antidiabetic agents on the risk of dementia. In comparison with no treatment for diabetes, the risk for dementia in the metformin users was decreased in the network meta-analysis (HR 0.75 95% CI 0.63–0.86) and in the separate meta-analysis (HR 0.86 95% CI 0.74–1.00) [110].

The authors concluded that patients treated with dipeptidyl peptidase-4 (DPP-4) inhibitors presented with the lowest risk of dementia, followed by those treated with metformin and thiazolidinedione, while treatment with insulin was associated with the highest risk. This might be related to the risk of hypoglycemia in insulin users, while this risk is very low in DDP-4 and metformin treatment.

One of the recent studies is the “Sydney Memory and Ageing Study” [111]. This prospective observational study is one of Australia’s largest and longest running studies of ageing and cognitive health. Of the 1037 patients, 91 were diagnosed with dementia during the 6-year follow up. Metformin use was associated with a decreased risk of dementia compared with non-metformin-using T2DM patients (HR 0.19 95% CI 0.04–0.85) and a non-significant different risk compared to non-diabetes patients.

Apart from prevention, metformin might also be beneficial in the treatment of established cognitive disease. Two small, randomized trials were performed with metformin in which verbal memory, executive function and cerebral blood flow were improved vs. placebo [112,113].

Taken together the evidence for a beneficial effect of metformin on cognitive function is not univocal. However, most studies do point to a protective effect of metformin on the risk of cognitive function and the risk of dementia in T2DM patients (Table 5). Compared with insulin and sulfonylurea, metformin has stronger beneficial effects possibly because of the lower risk of hypoglycemia.

Apart from a possible beneficial effect, there seems also a risk for a harmful effect of metformin regarding the increased rate of Parkinson’s disease. This might be confounded by an underlying vitamin B12 deficiency. It is known that long term metformin use can lead to vitamin B12 deficiencies [114]. B12 deficiency is related to increased disease progression in PD [115]. In future metformin studies related to cognitive function, it is vital to secure adequate B12 suppletion and/or to prevent B12 deficiencies.

## 6. Conclusions

Metformin is still the most commonly prescribed drug for the treatment of T2DM although new drug classes are competing for its leading position. Although the evidence for cardiovascular protection is stronger for the new drug classes than for metformin, it would be premature to discard metformin in this respect.

Combined with SGLT-2 inhibitors or GLP-1 receptor agonists, metformin has a favorable safety profile without increasing the risk of hypoglycemia. It might offer additional benefits, and therefore deserves to keep its foundational position, either in combination therapy in high cardiovascular risk T2DM patients or as first-line monotherapy in lower-risk patients.

The unique properties of metformin arising from its effects on cellular energy metabolism and oxidative stress extends its effects from cardiovascular protection to age-related diseases, such as cancer and dementia. Over and above an insulin-sparing effect, metformin distinguishes itself from other antidiabetic drugs with added value. Recent meta-analyses show a decrease in cancer incidence, an increase in cancer specific survival and a decrease in the incidence of dementia in T2DM patients.

Although most evidence in this field is observational, there is a growing number of randomized trials. A century after its chemical invention, metformin still deserves an important place, first and foremost in the treatment of T2DM, but possibly even in the larger non-diabetic population.

## Figures and Tables

**Table 1:** Mode of action of metformin in various physiological mechanisms.

Physiological Mechanism	Molecular Mechanism	Target Tissue
**Glucose metabolism**		
Decreased gluconeogenesis	Allosteric enzyme inhibition, redox state, complex 1 inhibition, AMPK activation	Liver [12]
Increased glucose uptake muscles	Increase in glucose transporters by SHIP2 inhibition	Skeletal muscles [22]
Increased gastrointestinal glucose uptake	Mitochondrial inhibition	Enterocyte [21]
GLP-1 secretion	AMPK	Enteroendocrine L cell [16]
**Vascular**		
Anti-inflammatory	Decrease NFkB	Macrophage [29]
Endothelial NO increase	AMPK	Endothelial cell [30]
Decrease oxidative stress	Mitochondrial ion channels and AMPK	Endothelial cell [31]
Hemostasis and leucocyte adhesion	Decrease in vWf and sVCAM-1	Endothelial cell [32]
Alternative energy substrate	Elevated lactate	Cardiomyocytes [33]
Decrease oxidative stress	AMPK and PP2A	Cardiomyocytes [25,26]
**Antineoplastic**		
Antiproliferation	Decrease mTOR	Tumor cells [34]
	Decreased insulin and free IGF-1 fraction	Tumor cells [34,35]
Reducing estrogen	Inhibition aromatase activity	Estrogen sensitive tumors [36]
Inhibition of glycolysis	Hexokinase2 inhibition	Tumor cells [37]
Decrease inflammation	Decrease NFkB	Macrophage [29]
Decrease antioxidative stress	Mitochondrial ion channels	Epithelial cells [28]
Inhibition of mesenchymal transition	Mitochondrial binding of copper	Tumor cells [38]
Improving immune response	AMPK and AMPK independent changes in tumor microenvironment	Immune cells [39]
**CNS protection**		
Decrease antioxidative stress	Mitochondrial	Microglia and neurons [24,40]
Increase insulin sensitivity	AMPK	Neurons [41]
Dephosphorylation proteins	AMPK and PP2A	Neurons [42]
Increase autophagy	Decrease mTOR	Hippocampus microglia [43,44]
Decrease inflammation	Decrease NFkB	Neurons [29]

vWf—von Willebrand factor; sVCAM-1—soluble vascular adhesion molecule-1; NO—nitric oxide; NFkB—nuclear factor kappa B; SHIP2—SH2-containing 5′-inositol phosphatase 2; PP2A—protein phosphatase 2; mTOR—mammalian target of rapamycin; IGF-1—insulin-like growth factor 1.

**Table 2:** Effect of metformin on cardiovascular outcomes in randomized trials.

Study (Reference)	Participants	Comparator	Endpoint	RR/HR/OR (95% CI)
Individual Trial				
UKPDS 1998 [61]	Newly diagnosed T2DM, *n* = 753	Diet	All-cause mortality	0.64 (0.45–0.91)
			Any diabetes related endpoint	0.68 (0.53–0.87)
UKPDS 2008 [3]	Newly diagnosed T2DM, *n* = 753	Diet	All-cause mortality	0.73 (0.59–0.89)
			Any diabetes related endpoint	0.79 (0.66–0.95)
HOME 2009 [62]	Insulin using T2DM, *n* = 390	Placebo	Macrovascular aggregate score	0.61 (0.40–0.94)
SPREAD-DIMCAD 2013 [63]	T2DM with coronary artery disease, *n* = 304	Sulfonylurea	Macrovascular aggregate score	0.54 (0.30–0.90)
**Meta analysis**	**Trials**			
Lamanna 2011 [57]	10	No therapy, placebo, active comparators	All-cause mortality	1.10 (0.80–1.51)
	12		Cardiovascular events	0.94 (0.82–1.07)
Boussageon 2012 [58]	11	Diet, placebo, no treatment, metformin add-on, metformin withdrawal	All-cause mortality	0.99 (0.75–1.31)
	10		Myocardial infarction	0.90 (0.74–1.09)
Griffin 2017 [59]	13	Diet, lifestyle, placebo	All-cause mortality	0.96 (0.84–1.09)
	7		Myocardial infarction	0.89 (0.75–1.06)
Monami 2021 [64]	13	Placebo/no therapy, active comparators	All-cause mortality	0.80 (0.60–1.07)
	2		MACE	0.52 (0.37–0.73)

MACE—major adverse cardiovascular event; RR—risk ratio; HR—hazard ratio; OR—odds ratio; CI—confidence interval.

**Table 3:** Effect of T2DM on cancer mortality and incidence.

Mortality	T2DM Relative Risk (95% CI)
Breast cancer	1.24 (0.95–1.62)
Colorectal cancer	1.20 (1.03–1.40)
Endometrial cancer	1.23 (0.78–1.93)
Hepatocellular carcinoma	2.43 (1.67–3.55)
Total cancer mortality	1.16 (1.04–1.30)
**Incidence**	
Breast cancer	1.20 (1.12–1.28)
Colorectal cancer	1.27 (1.21–1.34)
Endometrial cancer	1.97 (1.71–2.27)
Hepatocellular carcinoma	2.31 (1.87–2.84)
Intrahepatic cholangiocarcinoma	1.97 (1.57–2.46)
Pancreatic cancer	1.95 (1.66–2.28)
Total cancer incidence	1.10 (1.04–1.17)

Relative risk compared to non-diabetic individuals. Summary random effects estimate from 27 meta-analyses derived from Tsilidis et al. [77].

**Table 4:** Effect of metformin vs. active comparator in T2DM patients on cancer incidence and mortality.

Study (Reference)	Measure	Number of Cases	Breast Cancer	Colorectal Cancer	Lung Cancer
Tseng 2014 [81]	Incidence	~500k	0.63 (0.60–0.67)		
Libby 2009 [78]	Incidence	~8000	0.60 (0.32–1.10)	0.60 (0.38–0.94)	0.70 (0.43–1.15)
Chen 2017 [82]	Incidence	~45k	0.86 (0.70–1.05) ER− 1.25 (0.84–1.88) ER+		
Hui 2021 [83]	Mortality OS *	~4000	0.39 (0.25–0.60)		
Wang 2021 [84]	Incidence	~2 million		0.71 (0.64–0.80)	
	Mortality OS			0.72 (0.62–0.83)	
	Mortality CRC specific			0.80 (0.70–0.92)	
Xiao 2020 [85]	Incidence	~200k			0.78 (0.70–0.86)
	Mortality OS				0.65 (0.55–0.77)

Hazard ratio (95% confidence interval); OS—overall survival; CRC—colorectal cancer; ER—estrogen receptor; *—compared to non-diabetic individuals.

**Table 5:** Effect of metformin on cognitive function and dementia.

Study (Reference)	Participants	Comparator	Endpoint	OR/HR (95% CI)
**Individual trial**				
Samaras 2020 [111]	*N* = 1037	Non-metformin T2DM treatment	Dementia	0.19 (0.04–0.85)
**Meta analysis**	**Trials**			
Campbell 2018 [107]	3	Non-metformin T2DM treatment	Cognitive impairment	0.55 (0.38–0.78)
	6		Dementia	0.76 (0.60–0.97)
Ping 2020 [108]	23	Non-metformin T2DM treatment	Overall neurodegenerative disease	1.04 (0.92–1.17)
	17		Dementia	0.96 (0.85–1.08)
	3		Parkinsons disease	1.66 (1.14–2.42)
Zhou 2020 [110]	9	Diet	Dementia	0.75 (0.63–0.86)
	14		Dementia	0.86 (0.74–1.00)

OR—odds ratio; HR—hazard ratio; CI—confidence interval; T2DM—type 2 diabetes mellitus.

## Data Availability

Not applicable.
